# Match performance of football teams in different competition phases: Analysis on the data of eight consecutive seasons in the Chinese Super League

**DOI:** 10.3389/fpsyg.2022.1069082

**Published:** 2023-01-11

**Authors:** Pei Li, Shisheng Zhong, Paweł Chmura, Hongyou Liu

**Affiliations:** ^1^School of Physical Education & Sports Science, South China Normal University, Guangzhou, China; ^2^National Demonstration Centre for Experimental Sports Science Education, South China Normal University, Guangzhou, China; ^3^Department of Team Games, Wroclaw University of Health and Sport Sciences, Wroclaw, Poland

**Keywords:** notational analysis, match analysis, seasonal variability, soccer, association football, performance analyse

## Abstract

The study aims to quantify the variation in the physical and technical match performance of football teams in different months of a season in the Chinese Super League (CSL). Data of 1,899 matches in the seasons 2012–2019 of CSL collected by Amisco Pro® were analysed. The generalised mixed modelling was employed to estimate the per match mean values of six physical performance-related parameters and 16 technical performance-related parameters of CSL teams in every month of all the eight seasons. Results showed that: (1) the mean values of all the analysed physical performance-related parameters (total/sprint/HSR/MSR distance, sprint/HSR efforts) of CSL teams through a season were characterised like a ‘U’ shape, the highest value was observed in the beginning of season (March), then decreased gradually, reaching the lowest in August, and rebounded progressively from September to November; (2) the mean values of eight technical performance-related parameters (goals, shots, shot accuracy, individual possession, individual possession in the last third, crosses, cross accuracy and yellow cards) presented trivial changes through the whole season; (3) the number of passes, passes per shot, forward passes, and time in individual possession showed trivial changes from March to October, but showed a substantially increase in November (the last month of season); (4) Pass accuracy, forward pass accuracy, and the number of mean ball touches per individual possession substantially increased in June, July and August, whilst the number of challenges, ground challenges, air challenges, tackles and fouls all substantially decreased in these 3 months. These results could provide detailed information to help the practitioners choose the best training and match preparation strategy in the means of periodisation in different season phases.

## Introduction

A competitive season of professional football generally lasts for 9–10 months, and it is essential for the teams to maintain a stable (and in the best situation, the optimal) performance across an entire season to ensure an ideal end-of-season rank (Chmura et al., 2019). However, both physical and technical match performance of football players are facing a match-to-match variation (Rampinini et al., 2007; Bush et al., 2015; Carling et al., 2016; Liu et al., 2016a). Hence, the quantification of the seasonal variation of the match performance of football teams is of importance for coaches to adjust training programmes to maximise the physical and technical performance of the players and teams over the long-term competition and training cycle throughout an entire season (Chmura et al., 2019).

Initial investigation has been conducted by Mohr et al. (2003) and Rampinini et al. (2007), who both analysed the match running performance of Italian Serie-A players, and both concluded that players covered more total distance and high-intensity running distance in the end of season than in the start or in the middle of season. However, later studies analysing the data of players from the English Championship League indicated that the distance covered in sprinting and high intensity running were highest in matches of early-season and significantly decreased at mid-season which remained until late-season (Morgans et al., 2014b). Whilst more recently, Springham et al. (2020) divided an English Championship League into four quarters, and found that total distance, high-speed running and sprinting distance covered by players were all the highest in the matches of the first quarter of season. Meanwhile, Chmura et al. (2019) divided the *Bundesliga* (Germany first league of football) season into six periods, and found that total distance covered by the players peaked in the 4th period (two-thirds) of the season and decreased in the 5th and 6th period. Their study also included a comparison on the technical match performance of players during different phases of a season, and their results showed that the time of ball possession and number of passes achieved by players in matches of the 1st period of the season were lower than the 2nd period, whilst other technical performance showed no significant differences in consecutive periods of the season (Chmura et al., 2019).

As can be readily seen, all the above authors divided a competitive season intentionally and roughly into 3, 4 or 6 phases rather than classifying a season by the month of year. Morgans et al. (2014b) compared the physical and technical match performance in different months of a season using the data of players from the English Championship League. But due to the limitation of sample size (11 players from a single club), most of the comparisons are non-significant. Therefore, further analysis from this perspective using an amplified sample is warranted.

Furthermore, it is not difficult to find that existing research at this point are analysing the seasonal variation of performance at individual player level. However, due to the nature of match performance data, the value of some key performance indicators of players is always zero or missing (e.g., for the defensive players, the number of shots would often be zero, hence, the accuracy of shot is missing), so that could hardly be included in the analysis of player level. Thus, analyses at the team level would better allow a mixed-variable (combining all the possible physical and technical performance-related variables) design, which could provide deeper understanding of the football match performance (Bush et al., 2015). Physical performance of professional football is routinely analysed using the variables of total distance covered and distance covered in different intensities, whilst technical performance is regularly examined employing the goal scoring related parameters (number of shots and shot accuracy), passing and organising related parameters (number of passes and individual possessions) and defending related parameters (number of tackles and interceptions; Sarmento et al., 2014; Carling et al., 2016; Zhou et al., 2019).

Another issue that should be considered is that football match performance is documented to be influenced by situational variables such as game location (playing at home/away), match status/outcome (winning/win, drawing/draw, losing/loss), quality of team and opposition team, and so on (Taylor et al., 2008; Lago-Penas, 2012; Gómez et al., 2013; Sarmento et al., 2014). Specifically, football teams are performing higher numbers of goal scoring, passing and organising related technical actions whilst committing fewer fouls and receiving fewer cards in home games comparing to away games (Sarmento et al., 2014; Liu et al., 2015). Higher ranked teams generally involved more in actions with ball possession (Liu et al., 2015; Trewin et al., 2017), and covered more distance and high-speed-running distance whilst in ball possession (Hoppe et al., 2015; Trewin et al., 2017). Playing against stronger opposition demanded a higher level of technical, tactical and physical performance (Taylor et al., 2008; Castellano et al., 2011; Lago-Penas and Lago-Ballesteros, 2011; Liu et al., 2015). In elite football leagues, teams that won the match made more shots and shots on goal and performed fewer high-intensity activities than teams that did not win (Lago-Penas et al., 2010, 2011; Sarmento et al., 2014; Liu et al., 2015). Hence, confounding effects from these factors should be controlled when analysing the change in the performance of matches in different season phases (Springham et al., 2020).

On the other hand, the Chinese Super League (CSL) would serve as an ideal model for the analysis of rising professional football leagues, as it has attracted huge investment in the last years and recruited a large number of high-level players and coaches who may have brought in the latest match approaches and tactical concepts (Zhou et al., 2018, 2019). Along with that, the schedule of the CSL is normally within the same year, which is different from the major European leagues that are often with cross-year schedules.

Consequently, the current study aims to analysing the possible changes in the physical and technical match performance of football teams in different months of a season in the CSL using an amplified sample and controlling the effects of game location, match result, team and opponent strength. Based on previous research, we hypothesise that the physical performance would decline at the last months of the season, whilst the technical performance would keep relatively consistent throughout entire season.

## Materials and methods

### Sample and variables

Match performance statistics of 1,899 games from 2012 to 2019 in the CSL were analysed. Original data were collected by a semi-automatic computerised video tracking system, Amisco Pro®, whose working process, accuracy, validity and reliability have been discussed in detail in prior studies (Di Salvo et al., 2007; Carling et al., 2008; Andrzejewski et al., 2014). In line with the previous literature (Carling et al., 2016; Mao et al., 2016; Zhou et al., 2019), six physical performance-related parameters and 16 technical performance-related parameters (per team per match values) were chosen as dependent variables in the analysis. The grouping and definition of these variables are listed in Table 1. Competition phase (month of the season) was chosen as the main predictor variable, meanwhile, other four situational variables (match result, game location, team strength and opponent strength) were added as predictor variables as well. Ethics committee approval of this study was gained from the School of Physical Education & Sports Science of the South China Normal University (19CTY014).

**Table 1:** Selected technical and physical performance-related parameters (dependent variables).

*Physical performance-related parameters (unit): operational definition*
Total distance (m): Distance covered in a match by all the players of a team.
Sprint distance (m): Distance covered at the speed over 25 km/h in a match by all the players of a team.
HSR distance (m): Distance covered at high-speed-running (19.7–25 km/h) in a match by all the players of a team.
MSR distance (m): Distance covered at moderate-speed-running (14.3–19.6 km/h) in a match by all the players of a team.
Sprint efforts: Number of sprints in a match by all the players of a team.
HSR efforts: Number of high-speed-running in a match by all the players of a team.
*Technical performance-related parameters (unit): operational definition*
Goals: Number of goals scored by a team.
Shots: Attempts to score a goal, made with any (legal) part of the body, either on or off target.
Shot accuracy (%): Shots on target as a proportion of total shots.
Passes per shot (AU): The total number of passes divided by the total number of shots.
Individual possessions: Action begins when a player receives the ball and ends with a final ball contact before a non-neutral ball touch by another player.
Time in individual possession (min): Summed duration of all the individual possessions of all players of a team in a match.
Individual possessions in the last third: Individual possessions achieved in the attacking third of the pitch.
Mean ball touches per individual possession: Mean number of ball touches of all the individual possessions made by all the players of a team.
Passes: Intentional played balls from one player to another.
Pass accuracy (%): Successful passes as a proportion of total passes.
Forward passes: Intentional played balls from one player to another who is located in opponent’s half of pitch.
Forward pass accuracy (%): Successful forward passes as a proportion of total forward passes.
Cross: Balls sent into the opposition team’s area from a wide position.
Cross accuracy (%): Successful crosses as a proportion of total crosses.
Challenges: Actions when two players are competing for the possession of a ball which is not in control by any player, both players have roughly 50% chance of gaining control of the ball. Including ground and air challenges.
Ground challenges: Two players challenge each other competing for the possession of a ball which is below hip height of the player who touched the ball.
Air challenges: Two players challenge each other competing for the possession of a ball which is above hip height of the player who touched the ball.
Tackles: Players attempting to get the ball from another player who is in control of the ball.
Fouls: Any infringement that is penalised as foul play by a referee.
Yellow cards: Where a player was shown a yellow card by the referee for reasons of foul, persistent infringement, hand ball, dangerous play and time wasting.

Parameters without unit are counts.

### Procedure and statistical analysis

The generalised mixed linear model was attributed to be one of the best solutions to properly account for the repeated-measures problem of multiple games played by single teams in professional football leagues (Liu et al., 2016b), hence was chosen as the modelling tool in this study. The modelling was realised with Proc Glimmix in the University Edition of Statistical Analysis System (version SAS Studio 3.6). Separate Poisson regressions were run in the model taking the value of each of the six physical and 16 technical performance-related parameters as the dependent variable. A random effect for team identity was used to account for repeated measurement on the teams. The fixed effects estimated the effects of the five predictor variables (competition phase, match result, game location, team strength and opponent strength).

Competition phase, match result and match location were included as nominal variables. Competition phase was with nine levels (named 3–11, stands for the months of March through November). Match result was with three levels (named 3, 1 and 0, stands for win, draw and loss). Game location was with two levels (named 1 and 2, stands for home and away). The effect of team strength and opponent strength was estimated by adding a new predictor (Phillips and Hopkins, 2017; Zhou et al., 2019), which is ‘strength_diff = log (team rank/opponent rank).’

The established models can estimate the mean values of each of the physical and technical performance-related parameters in each month, controlling the effects of match result, game location, team strength and opponent strength. Estimated mean values of each of the parameters in the months from April to November were compared to the value of March to get the mean changes. Uncertainty in the true changes was evaluated using the non-clinical magnitude-based inference (Hopkins et al., 2009) as implemented in the spreadsheet accompanying the package of materials for generalised mixed modelling with SAS Studio (Hopkins, 2016). Observed magnitudes and their confidence limits were expressed in standardised units, whereby the change in means was divided by the observed between-match standard deviation (SD) derived from the mixed model, and then evaluated qualitatively with the following scale: <0.2 trivial, 0.2–0.6 small, 0.6–1.2 moderate, 1.2–2.0 large, >2.0 very large. Effects were deemed clear if the 99% confidence interval did not include positive and negative substantial values simultaneously. Clear effects were reported with a qualitative likelihood that the true difference was either substantial or trivial (whichever probability was greater) using the following scale: <0.5% most unlikely, 0.5–5% very unlikely, 5–25% unlikely, 25–75% possibly, 75–95% likely, 95–99.5% very likely, >99.5% most likely.

## Results

### Physical performance of teams at different competition phase

**Figure 1:**
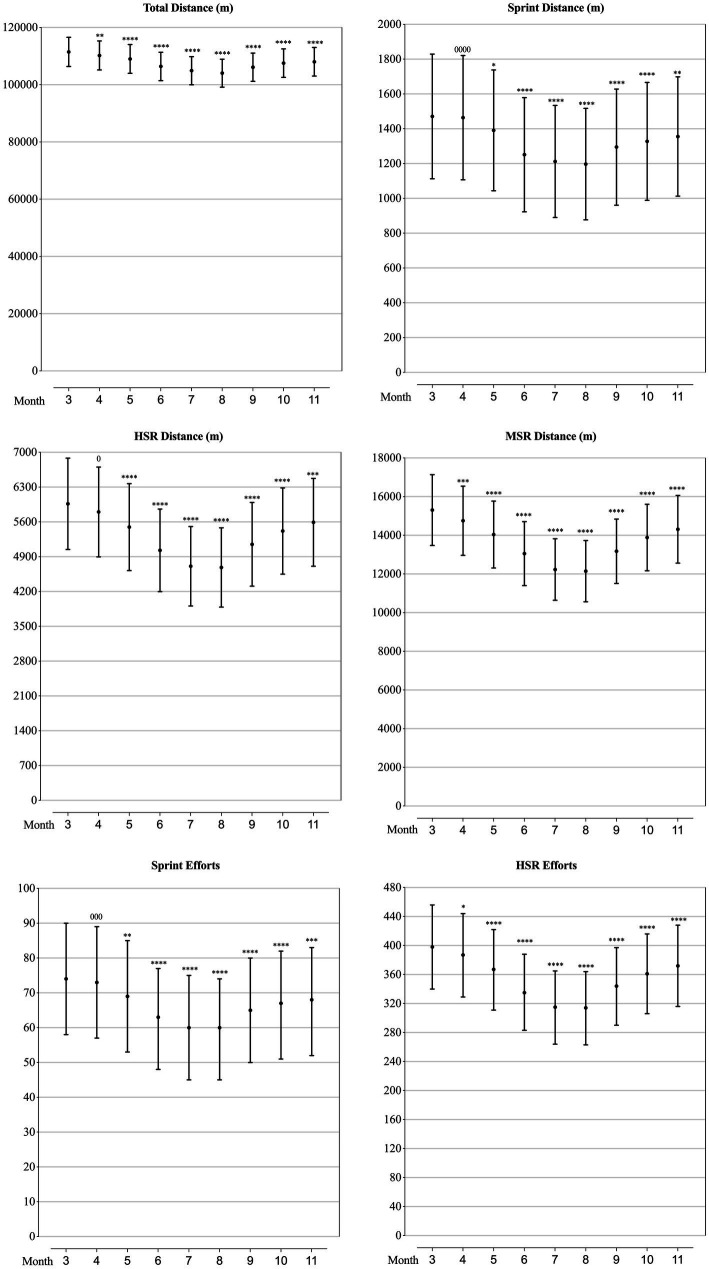
Values of physical performance-related parameters in each month of a season for the teams competing in the CSL in 2012-2019 estimated from the generalised mixed linear modelling. Filled dots are the means. Error bars are the standard deviations. Symbols “0” and “*” stands for trivial and negative change from the value of March (month “3”). Number of symbols indicate the likelihood for the magnitude of the true effect as follows: 1-possible; 2-likely; 3-very likely; 4-most likely.

As can be seen from Figure 1, all the analysed physical performance-related parameters showed a similar variation trend throughout a season, which is characterised like a ‘U’ shape. The highest values occurred in March, teams achieved the highest values in Total Distance, Sprint Distance, HSR Distance, MSR Distance, Sprint Efforts and HSR Efforts. Whilst the values started to decrease substantially in April or May, and reached the lowest in August. And in the late season (September–November), the values gradually went up, but could hardly rebound to the values of March.

### Technical performance of teams at different competition phase

**Figure 2:**
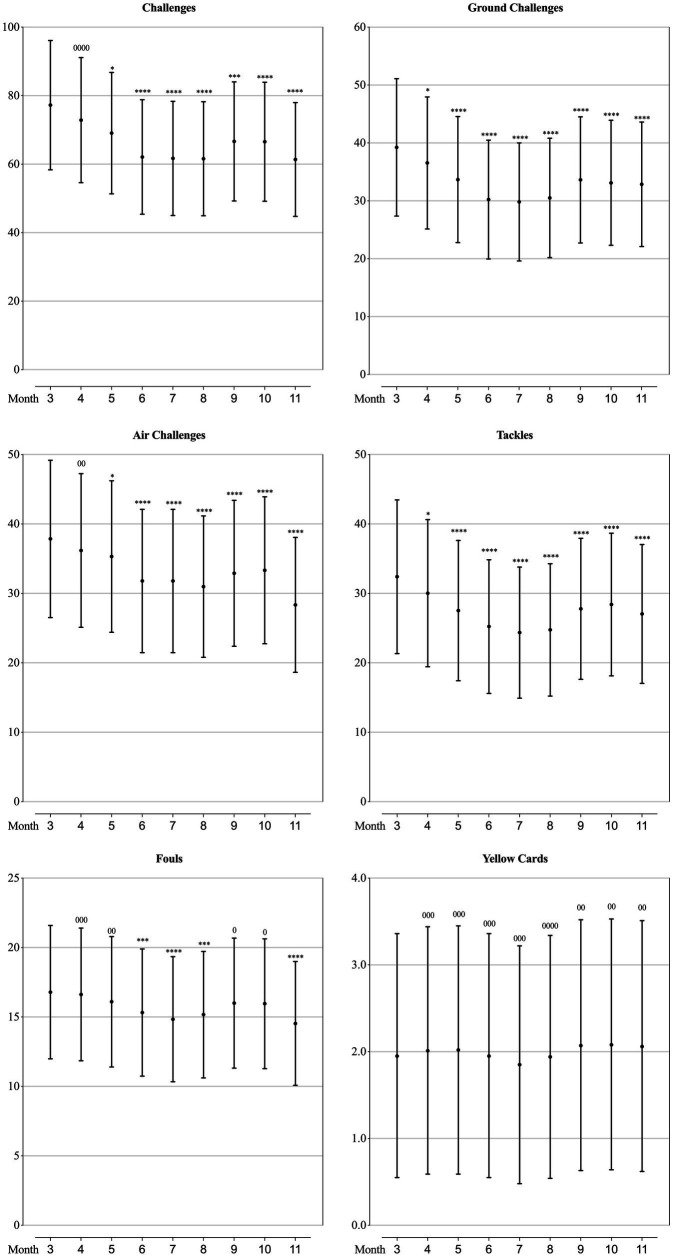
Values of goal-scoring related parameters in each month of a season for the teams competing in the CSL in 2012-2019 estimated from the generalised mixed linear modelling. Filled dots are the means. Error bars are the standard deviations. Symbols “0” and “*” stands for trivial and negative change from the value of March (month “3”). Number of symbols indicate the likelihood for the magnitude of the true effect as follows: 1-possible; 2-likely; 3-very likely; 4-most likely.

**Figure 3:**
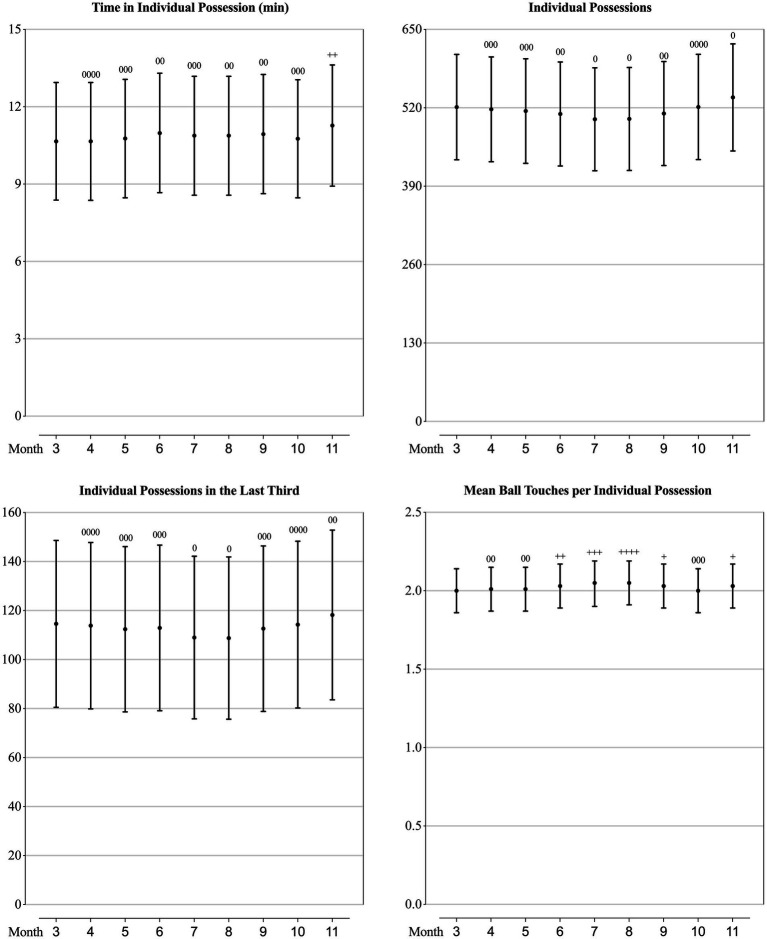
Values of ball possession-related parameters in each month of a season for the teams competing in the CSL in 2012-2019 estimated from the generalised mixed linear modelling. Filled dots are the means. Error bars are the standard deviations. Symbols “0” and “+” stands for trivial and positive change from the value of March (month “3”). Number of symbols indicate the likelihood for the magnitude of the true effect as follows: 1-possible; 2-likely; 3-very likely; 4-most likely.

**Figure 4:**
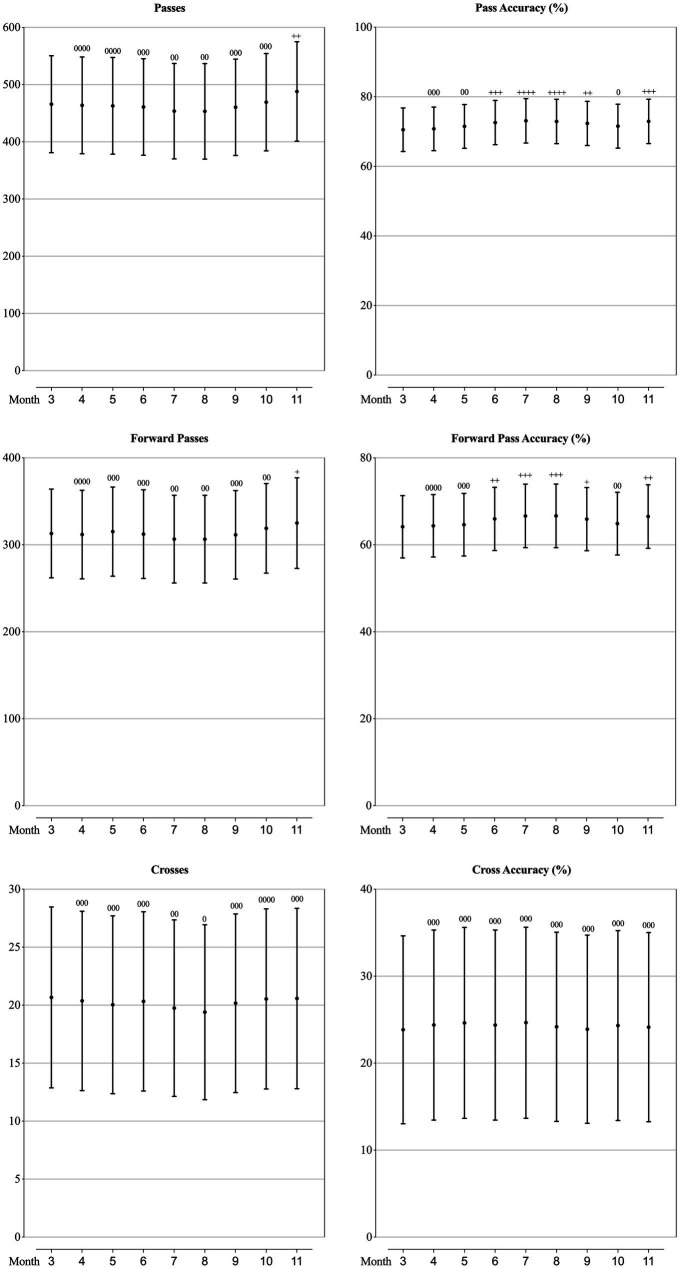
Values of passing-related parameters in each month of a season for the teams competing in the CSL in 2012-2019 estimated from the generalised mixed linear modelling. Filled dots are the means. Error bars are the standard deviations. Symbols “0” and “+” stands for trivial and positive change from the value of March (month “3”). Number of symbols indicate the likelihood for the magnitude of the true effect as follows: 1-possible; 2-likely; 3-very likely; 4-most likely.

**Figure 5:**
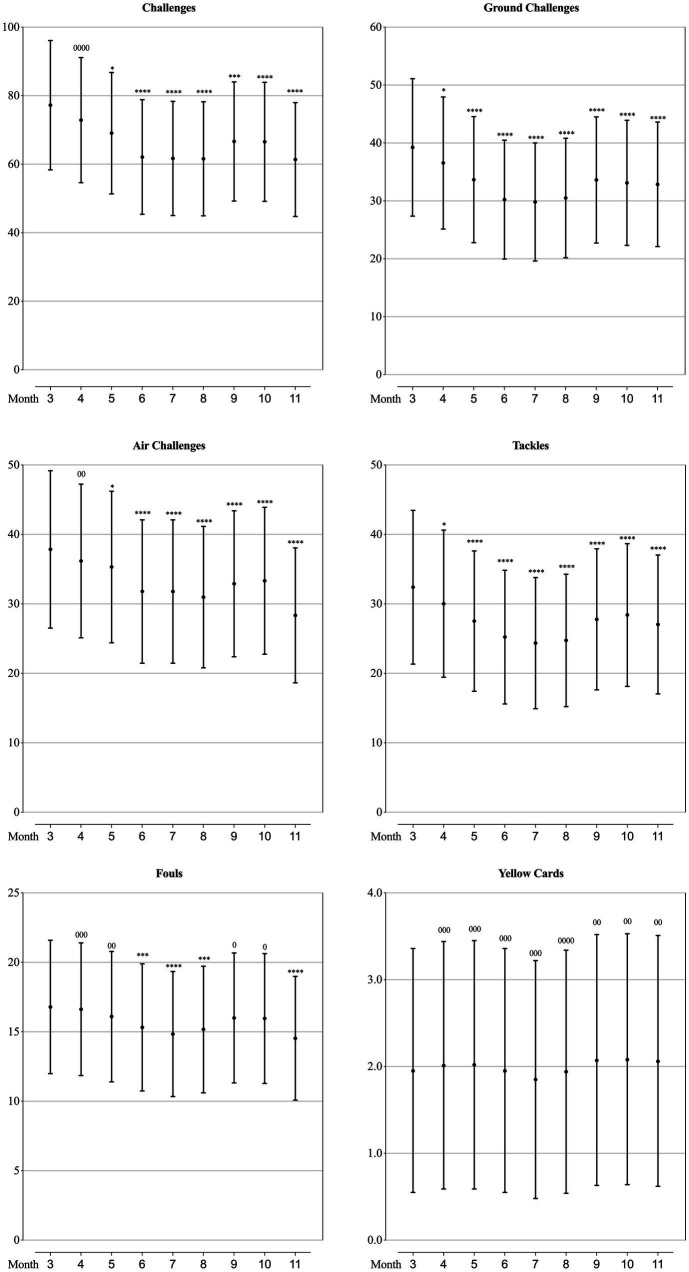
Values of defending-related parameters in each month of a season for the teams competing in the CSL in 2012-2019 estimated from the generalised mixed linear modelling. Filled dots are the means. Error bars are the standard deviations. Symbols “0” and “*” stands for trivial and negative change from the value of March (month “3”). Number of symbols indicate the likelihood for the magnitude of the true effect as follows: 1-possible; 2-likely; 3-very likely; 4-most likely.

As can be seen from Figures 2–5, the mean values of eight technical performance-related parameters (goals, shots, shot accuracy, individual possession, individual possession in the last third, crosses, cross accuracy and yellow cards) presented trivial changes through the whole season. The number of passes, passes per shot, forward passes and time in individual possession showed trivial changes from March to October, but showed a substantially increase in November. Pass accuracy, forward pass accuracy, and the number of mean ball touches per individual possession substantially increased in June, July and August, whilst the number of challenges, ground challenges, air challenges, tackles and fouls all substantially decreased in these 3 months.

## Discussion

Using a long-term spanned dataset, the current study aims to quantify the changes in the physical and technical match performance of football teams in different competition phase of a season in the CSL, in order to determine the seasonal variation of the match performance of professional football.

In prior studies, the physical performance of football players in matches of different competition phases have been investigated, but controversy results have been found. Early research (Mohr et al., 2003; Rampinini et al., 2007) indicated that players covered the most total distance and high-intensity running distance in the matches of the end season, whilst later studies showed that the distance covered in sprinting and high intensity running were highest in matches of early-season (Morgans et al., 2014b; Springham et al., 2020). What is more, recent evidence showed that physical performance of football players reached to the peak at the two-thirds of the season (Chmura et al., 2019). Distinctly to any of the prior studies, our results showed that all the six analysed physical performance-related parameters (total/sprint/HSR/MSR distance, sprint/HSR efforts) of CSL teams showed a similar ‘U’-shaped variation trend through a competitive season: the highest value was observed in the early season (first month, March), then decreased gradually, reaching the lowest in the two-thirds of the season (sixth month, August), and rebounded progressively at the end of season (November). Accumulated longitudinal fatigue, short of recovery time, congested competition schedule, and mid-season breaks have been previously attributed to the cause of the seasonal variation in the physical match performance (Mohr et al., 2003; Morgans et al., 2014b; Chmura et al., 2019; Springham et al., 2020). Acknowledging the effects of these factors, we would further link this variation to the weather change. According to previous studies, in the *Bundesliga*, total distance covered and high intensity efforts made by players in matches decreased when the temperature increased (Chmura et al., 2021), whilst in the CSL, players achieved the highest values of physical performance-related parameters at the temperature between 10.6 and 22°C (Zhou et al., 2019). Meanwhile, the monthly average temperature in China showed a trend of rising first and then decreasing from March to November, and reaching the highest in July and August (Li and Zha, 2019), which perfectly coincided the trend of variation of the physical performance of CSL teams.

As mentioned previously, existing studies focusing on seasonal variation were investigating the performance at individual player level, therefore, limited technical performance-related variables were included. Chmura et al. (2019) analysed the seasonal variation in six technical performance-related parameters (ball possession time, number of passes, passing accuracy, number of one-on-one duels, number of one-on-one duels won, percentage of one-on-one duels won) of players in the *Bundesliga*, and found that the ball possession time and number of passes achieved by players in matches of the 1st period of the season were lower than the 2nd period, whilst other technical performance showed no meaningful changes in consecutive periods of the season, and hence concluded that players were capable to maintain their level of technical performance through the entire season. Analysing from the team perspective, we could have had more performance-related parameters contained. What is similar with the *Bundesliga* (Chmura et al., 2019), all the goal-scoring related parameters (goals, shots and shot accuracy), some ball possessing related parameters (individual possession, individual possession in the last third) and a discipline related parameter (yellow cards) of the CSL teams presented trivial changes through the whole season, which coincided the trend of inter-season variation between 2012 and 2017 in the CSL (Zhou et al., 2020). What is different from the *Bundesliga* (Chmura et al., 2019), we found that the passing-related and defending-related parameters of the CSL teams showed some meaningful variation in the season. Specifically, the number of passes, passes per shot, forward passes, and time in individual possession kept relatively stable from the 1st to the 8th month of the season, but substantially increased in the last month, meanwhile, the accuracy of pass and forward pass increased as well. Combining with the result that the number of challenges, air challenges, tackles and fouls decreased substantially in the last month, it is reasonable to infer that CSL teams are taking more cautious and conservative match strategies (trying the best to control the ball and reducing unnecessary dules and fouls) to strive for an acceptable match outcome in order to grab enough points to ensure an ideal ranking at the end of the season. Another worthwhile seasonal change is that the accuracy of pass and forward pass, the number of mean ball touches per individual possession substantially increased in June, July and August, but the number of challenges, ground challenges, air challenges, tackles and fouls all substantially decreased in this period. This trend should be attributed to the variation of the physical performance which showed the most decrease in these 3 months. It is believed that in football matches, when the team were out of ball possession and defending, the physical demands were greater, vice versa, when the team were in ball possession, the physical demands were lower (Morgans et al., 2014a; Castellano and Pic, 2019; Kong et al., 2022). Hence our results would indicate that in the most demanding months (June, July and August) in the CSL, teams are not capable to maintain their optimal physical performance, so that they tend to ‘slow down’ the temper of the matches by doing more ball touches and ensuring high passing accuracy, and reducing duels and fouls.

## Conclusion

The physical match performance of the CSL teams showed a ‘U’-shaped variation trend through a competitive season: the highest value was observed in the early season, then decreased gradually, reaching the lowest in the middle (two-thirds) of the season, and rebounded progressively at the end of season. All the goal-scoring related parameters of the CSL teams presented trivial changes through the whole season. The number of passes, passes per shot, forward passes, and time in individual possession kept relatively stable in most months of the season, but substantially increased in the last month. June, July and August may have been the toughest months in the CSL, when teams were not capable to maintain their optimal physical performance, but tried to do more ball touches and to ensure higher passing accuracy, and to reduce duels and fouls.

### Practical application and limitation

Attention should be paid to the change in the running capacity of CSL players to generate the best training programmes to ensure the optimal physical condition in upcoming matches. In the middle of the season (July and August), the volume and intensity of training load should be specially adjusted to meet the match demands and to avoid potential injury.

One of the biggest shortcomings is that the study is just a post-match and post-season analysis, which took only the match outcome and end-of-season rank as the controlling factors. Future analysis should consider the match score-line and the live ranking in the league table when the match played. Furthermore, more research could be conducted to examine that if the ‘toughest months’ in the CSL are generated by the environmental factors (e.g., temperature).

## Data availability statement

The raw data supporting the conclusions of this article will be made available by the authors, without undue reservation.

## Ethics statement

The studies involving human participants were reviewed and approved by Ethics committee of the School of Physical Education & Sports Science, South China Normal University. The ethics committee waived the requirement of written informed consent for participation.

## Author contributions

PL was involved in data collection, drafted and revised the manuscript. SZ was involved in the data collection and interpretation. PC was involved in the data interpretation, drafted and revised the manuscript. HL developed the idea, was involved in the research design, data collection, analysis and interpretation, drafted and revised the manuscript. All the authors approved the final version of the manuscript.

## Funding

This work is supported by the National Social Science Fund of China (NSSFC) under grant number 19CTY014.

## Conflict of interest

The authors declare that the research was conducted in the absence of any commercial or financial relationships that could be construed as a potential conflict of interest.

## Publisher’s note

All claims expressed in this article are solely those of the authors and do not necessarily represent those of their affiliated organizations, or those of the publisher, the editors and the reviewers. Any product that may be evaluated in this article, or claim that may be made by its manufacturer, is not guaranteed or endorsed by the publisher.
